# Neurofibromatosis Type 1—Retinal Alterations Detectable with Optical Coherence Tomography Angiography

**DOI:** 10.3390/diagnostics14131447

**Published:** 2024-07-06

**Authors:** Anca Elena Târtea, Carmen Luminița Mocanu, Alin Ștefan Ștefănescu Dima, Andreea Cornelia Tănasie, Veronica Maria, Alexandra Oltea Dan, Andrei Theodor Bălășoiu

**Affiliations:** 1 Department of Neurology, University of Medicine and Pharmacy of Craiova, 200349 Craiova, Romania; 2 Department of Ophthalmology, University of Medicine and Pharmacy of Craiova, 200349 Craiova, Romania; 3 Department of Pediatrics, University of Medicine and Pharmacy of Craiova, 200349 Craiova, Romania

**Keywords:** neurofibromatosis type 1, retinal microvascular alterations, choroidal nodules, optical coherence tomography angiography

## Abstract

Neurofibromatosis type 1 (NF 1) is a multisystemic genetic disorder involving aberrant proliferation of multiple tissues of a neural crest origin. It represents a tumor predisposition syndrome characterized by a wide range of clinical manifestations, such as benign tumors, which primarily affect the skin and the nervous system. The most frequent clinical signs of NF 1 include café-au-lait spots all over the surface of the skin and axillary freckling; however, these signs can be accompanied by more severe manifestations such as the growth of both benign and malignant nervous system tumors and skeletal dysplasia, as well as a wide range of ocular manifestations. We report the rare case of retinal microvascular alterations and choroidal nodules in a 15 year old male patient with NF 1, detectable on optical coherence tomography angiography (OCTA). The hyperreflective choroidal nodules modified the profile of the choroidal vasculature. The retinal microvascular alterations in the form of clustered capillaries were detected in the superficial capillary plexus located nasally to the macular region. Retinal vascular abnormalities undetectable on fundus photography or fundoscopy can be present in patients with NF 1. Indirect ophthalmoscopy of our study patient was unremarkable. However, retinal vascular abnormalities were seen on OCTA scans in the superficial capillary plexus and choroidal nodules were detected on raster OCT scans. OCTA represents a useful imaging technique for detecting retinal microvascular abnormalities, which can be considered additional distinctive signs of NF 1.

## 1. Introduction

Neurofibromatosis type 1 (NF 1) is a multisystemic genetic disorder involving aberrant proliferation of multiple tissues of neural crest origin [1]. It represents a tumor predisposition syndrome, characterized by a wide range of clinical manifestations, the most specific being the benign tumors which primarily affect the skin and the nervous system [2]. Its prevalence is approximately 1 in 2500–3000 individuals worldwide, with complete penetrance around 5 years of age [3].

NF 1 is caused by a mutation on chromosome 17–17q11.2 encoding neurofibromin type 1, a cytoplasmatic protein with oncosuppressive activity [4]. Neurofibromin type 1 is a negative regulator of the RAS proto-oncogene, which is predominantly expressed in neurons, Schwann cells, oligodendrocytes and astrocytes. The lack of this protein leads to excessive cell growth and tumor formation [5]. Therefore, NF 1 is considered a RASopathy, being defined as a disorder caused by a germline mutation causing dysregulation of the RAS mitogen-activated protein kinase pathway [6].

NF 1 is transmitted by autosomal dominant inheritance in approximately 50% of individuals, but can also be caused by spontaneous mutations in patients without any family history of the disease [7]. The disease presents variable phenotypic expression and considerable clinical heterogeneity even within the same family members [8]. It also presents an unpredictable evolution with slowly, but progressively, growing neurofibromas with increased risk of malignant transformation [9]. The most frequent clinical signs of NF 1 include café-au-lait spots all over the surface of the skin and axillary freckling; however, these signs can be accompanied by more severe manifestations such as the growth of both benign and malignant nervous system tumors and skeletal dysplasia.

### 1.1. Clinical Diagnosis

For the clinical diagnosis of NF 1 to be established, at least two out of the seven following revised criteria need to be met. The diagnostic criteria currently used for diagnosis were presented by the National Institute of Health statement in 1987 and revised in January 2020 [10]:

- An amount of 6 or more café-au-lait macules over 5 mm in greatest diameter in pre-pubertal individuals and over 15 mm in greatest diameter in post-pubertal individuals;
- Freckling in the axillary or inguinal region;
- Two or more neurofibromas of any type or one plexiform neurofibroma;
- Optic pathway glioma;
- Two or more iris Lisch nodules identified by slit lamp examination or two or more choroidal abnormalities (CAs)—defined as bright, patchy nodules imaged by optical coherence tomography (OCT)/near-infrared reflectance (NIR) imaging;
- A distinctive osseous lesion such as sphenoid dysplasia, anterolateral bowing of the tibia or pseudarthrosis of a long bone;
- A heterozygous pathogenic NF 1 variant with a variant allele fraction of 50% in apparently normal tissue such as white blood cells.

### 1.2. Ocular Manifestations

Several ocular manifestations of NF 1 affect the eye and the periocular adnexa, and recent advancements in imaging techniques are now providing new insights to study the ophthalmic manifestations of the disease [11]. Characteristic ophthalmic lesions, such as Lisch nodules and optic gliomas, represent frequent clinical findings of the disease and are therefore considered diagnostic hallmarks [12].

Lisch nodules are pathognomonic markers of NF 1 and are seen on biomicroscopic examination as dome-shaped solid lesions on the iris’ surface [13]. Histologically, they consist of melanocytic hamartomas composed of melanocytes, elongated fibroblasts and mast cells. Their number and diameter increases with age and they have a bilateral appearance in most cases. Although they represent characteristic signs of NF 1, Lisch nodules are clinically asymptomatic and do not usually cause any visual disturbance [14].

Optic gliomas (OPGs) are low-grade benign tumors [15], classified as grade 1 pilocytic astrocytomas by the WHO (World Health Organization), which occur in approximately 15–20% of patients with NF 1 [16]. NF 1-OPGs are most frequently observed in children under the age of seven, but there have been rare instances of NF 1-OPGs emerging during adolescence or adulthood [17]. They can be localized anywhere along the visual pathway, at the optic nerve, chiasm, optic tracks and radiations [16]. The clinical manifestations of OPGs vary based on their size and location. When located in the orbit, they can lead to proptosis, papillary edema, reduced visual acuity and visual field defects [18]. Intracranial localization of OPGs result in specific neurological symptoms associated with mass-occupying lesions.

Palpebral plexiform neurofibromas are other ocular manifestations, traditionally considered pathognomonic for NF 1. They are characterized by unilateral upper eyelid localization and typically develop after 2 years of age. These lesions can cause loss of visual acuity due to asymmetric ptosis and alteration of the eyelid anatomy [19].

Other ocular manifestations of NF 1 include retinal astrocytic hamartomas, retinal capillary hemangiomas and choroidal alterations visible on optical coherence tomography OCT or NIR evaluation [20]. The choroidal nodules are formed by proliferating Schwann cells and have a similar histological origin as cutaneous neurofibromas and Lisch nodules [21]. Glaucoma has also been reported in patients with NF 1, with variable etiology such as ectropion uveae [22] or neovascular proliferation secondary to vasculopathy of the distal ophthalmic artery [23].

In recent years, highly performant, noninvasive imaging techniques, such as optical coherence tomography angiography (OCTA), have improved the visibility of deep retinal vascular abnormalities without using contrast agents. These specific retinal alterations would not be visible during conventional direct or indirect ophthalmoscopy.

Therefore, the aim of our ophthalmic examination of a patient newly diagnosed with NF 1 was to further investigate retinal and choroidal structures with the help of OCTA scanning.

## 2. Case Report

We report a rare case of vascular retinal alterations and choroidal nodules in a 15 year old male patient with NF 1. The patient was referred for ophthalmic examination after being newly diagnosed with NF 1 within the Pediatric Department of the Clinical Emergency Hospital, Craiova, Romania. The patient did not report any visual disturbances or any other ocular symptoms. Before presenting in our department, the patient did not receive any chronic systemic treatment. The patient’s family history was unremarkable for NF 1.

**Figure 1:**
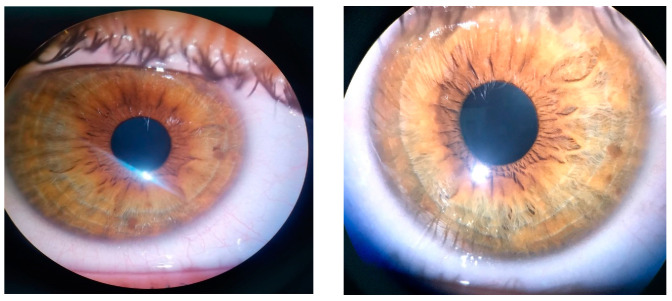
Lisch nodules visible in RE and LE.

The ophthalmic examination revealed a bilateral uncorrected visual acuity of 20/30 and 20/20 best corrected visual acuity, with a refraction of −0.50 spherical diopters in the right eye (RE) and −0.75 spherical diopters in the left eye (LE). The intraocular pressure was 12 mmHg (RE) and 15 mmHg (LE). On slit lamp examination, Lisch nodules could be seen bilaterally on the iris’ surface. There were 5 Lisch nodules detected in the RE and 3 Lisch nodules in the LE, ranging from 0.2 mm in diameter up to 0.7 mm in diameter (Figure 1). There were no other abnormalities detected on anterior segment examination in BE.

The dilated fundus examination in BE revealed a clear vitreous, contoured optic nerve disc with physiological cupping, without any retinal vascular abnormalities visible on indirect ophthalmoscopy.

**Figure 2:**
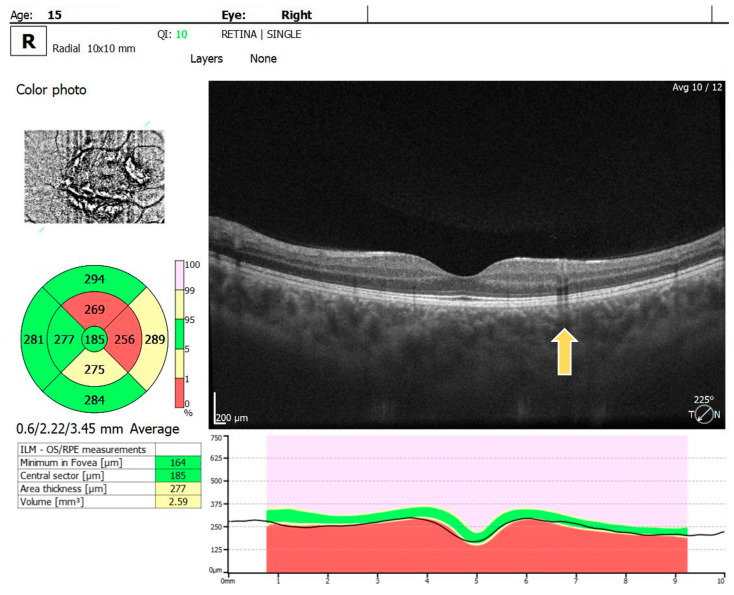
OCT scan of the RE: arrow showing a hyperreflective area in the nerve fiber layer and ganglion cell layer.

**Figure 3:**
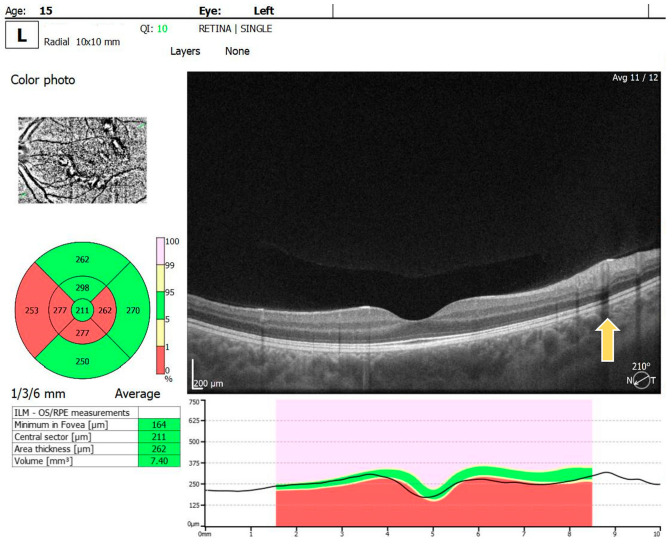
OCT scan of the LE: arrow showing a hyperreflective area in the nerve fiber layer and ganglion cell layer.

For the RE, the macula OCT investigation showed a slightly thin macula with a foveal thickness of 199 µ and a hyperreflective area nasally to the macula in the nerve fiber layer and ganglion cell layer (Figure 2). For the LE, the macula OCT showed a foveal thickness of 211 µ and the same aspect of a hyperreflective area nasally to the macula in the nerve fiber layer and ganglion cell layer (Figure 3).

**Figure 4:**
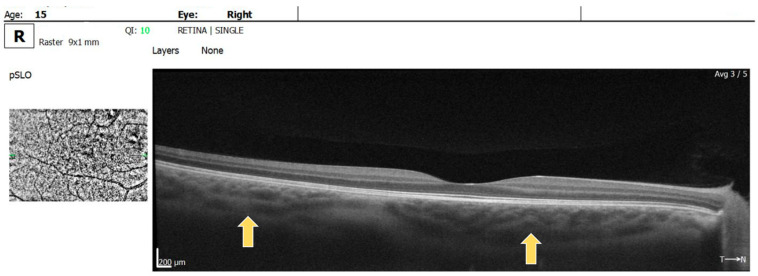

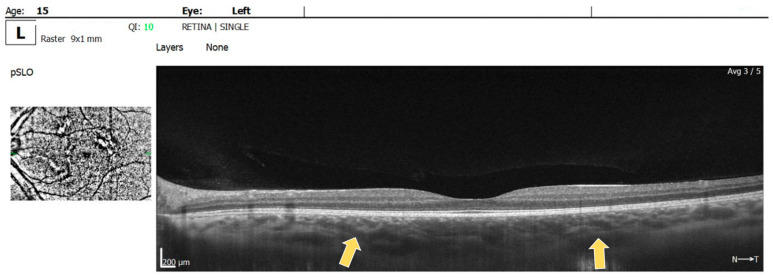
OCT raster: arrow showing choroidal nodules in BE.

Multiple hyperreflective choroidal nodules of various sizes were visible on OCT raster images in BE, expanding beyond the area of retinal microvascular abnormalities seen on the OCTA (Figure 4).

**Figure 5:**
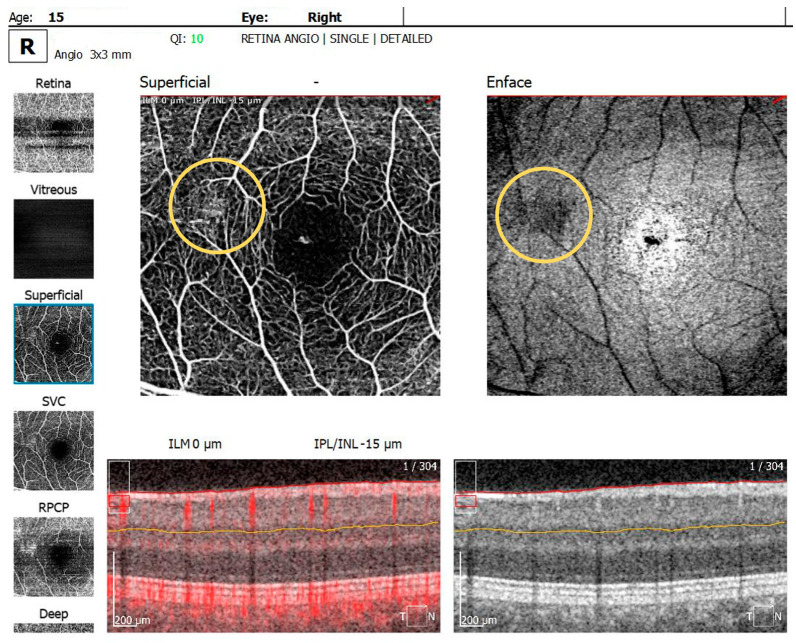

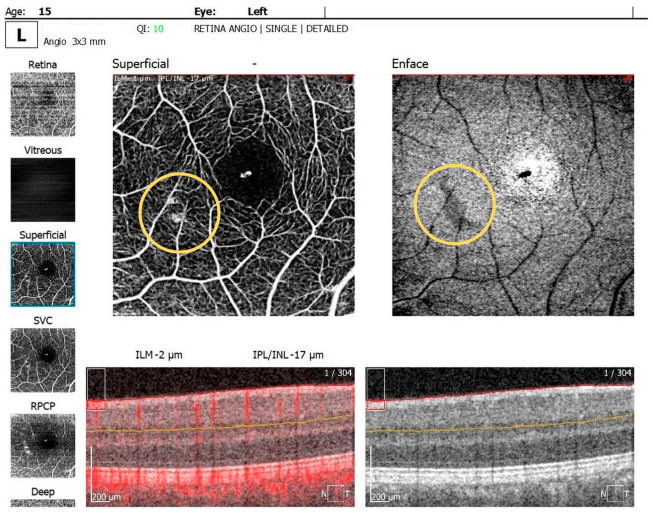
OCTA scan showing clustered capillaries visible in the superficial capillary plexus located nasally to the macular region in BE.

The OCTA examination revealed the following values for the foveal avascular zone (FAZ) parameters in the RE: the FAZ area was 0.49 mm^2^, the FAZ perimeter was 3.55 mm and the FAZ circularity index was 0.49. Retinal microvascular alterations in the form of clustered capillaries were visible in the superficial capillary plexus located nasally to the macular region (Figure 5). For the LE, the FAZ area was 0.47 mm^2^, the FAZ perimeter was 3.19 mm and the FAZ circularity index was 0.49; a similar area of clustered capillaries was visible in the superficial capillary plexus located inferonasally to the macula.

## 3. Discussion

The presence of choroidal nodules is a frequent finding in patients with NF 1 [24]. These nodules are not visible on fundus examination, but they are detectable on OCT or NIR examination, where they appear as multiple bright patches. The vascularization surrounding choroidal nodules is affected through hypoperfusion of the choroid [25]. On the other hand, retinal microvascular alterations are a rare finding, with insidious clinical presentation in patients with NF 1. Retinal microvascular alterations can be analyzed with the help of OCTA scanning, a cutting-edge imaging technique, which provides remarkable details of the retinal and choroidal vascular network without the use of contrast agents [26]. According to a large study conducted by Parrozzani Raffaele et al. [27] including 294 patients diagnosed with NF 1, these changes were present in only 6% of cases. The research by Parrozzani et al. showed similar retinal vascular changes in the superficial capillary plexus of their study patients. For the majority of their study patients, these vascular abnormalities were visible in the deep capillary plexus as well. In contrast with this finding, our investigated case did not show any vascular abnormalities in the deep capillary plexus, but we will monitor the patient and look for possible vascular changes in the deep capillary plexus in upcoming ophthalmic examinations.

Similarly to our report, Catherine Cassiman et al. also reported 3 cases of retinal microvascular changes in patients with NF 1 visible in the superficial capillary plexus; however, they do not mention the presence or absence of Lisch iris nodules in any of their patients.

The spectrum of retinal microvascular lesions in patients with NF 1 ranges from simple vascular tortuosity to the more complex corkscrew patterns and Moyamoya-like arrangements. The pathogenesis of these vascular lesions in NF 1 is currently being researched. One possible mechanism described by Antonietta Moramarco et al. [28] refers to neurofibromin, which contributes to the integrity of the endothelial cell layer. Consequently, the absence of neurofibromin in NF 1 patients may alter the interaction between endothelial cells and pericytes, providing stimulatory signals for both cell types to proliferate, leading to retinal microvascular alterations.

OCTA represents a noninvasive imaging technique that provides in-depth visualization of retinal and choroidal vasculature. This method combines angiographic and structural data to create a cube scan, from which en-face images of the vasculature at different axial positions can be extracted. OCTA has significantly enhanced our understanding of retinal vascular disorders and has been primarily utilized for the qualitative analysis of vascular structures. The OCTA technique has the ability to evaluate the retinal superficial capillary plexus and the deep capillary plexus separately, providing detailed three-dimensional information about early microcirculatory disturbances. OCTA is unique in offering the possibility of separately analyzing the retinal superficial capillary plexus (SCP), the deep capillary plexus (DCP) and the choroidal vasculature, and allowing a better understanding of pathophysiological changes in the retinal and choroidal vascular network of patients with NF 1 [29].

Retinal capillary clusters appear on OCTA as isolated formations within the superficial vascular plexus, and they are typically characterized by a dense network of capillaries distinct from the surrounding vasculature [30].

### Limitations of the Study

One limitation of the current study is that we describe a single ophthalmic examination of our patient, as this was a newly diagnosed NF 1 case. We will aim to regularly perform comprehensive ophthalmic examinations of our patient and to report any further changes, should they occur. Furthermore, our OCTA device enabled us to examine only the central part of the posterior pole; a wide-field OCTA could observe more parameters and provide a broader perspective.

## 4. Conclusions

Retinal vascular abnormalities undetectable on fundus photography or fundoscopy can be present in patients with NF 1. Indirect ophthalmoscopy of our study patient was unremarkable. However, retinal vascular abnormalities were seen on OCTA scans in the superficial capillary plexus and choroidal nodules were detected on raster OCT scans. Although these retinal and choroidal changes were not associated with ischemia, monitoring these changes can help track the progression of NF 1, providing valuable information about the disease’s impact on the patient over time.

OCTA has a valuable role in managing NF 1 patients, as it offers an objective, quantitative assessment of retinal microvasculature, without the need for a contrast substance, requiring only a few seconds of patient cooperation. OCTA has proven to be a useful tool for detecting retinal microvascular abnormalities, which can be considered an additional distinctive sign of the disease in patients affected by NF 1.

## Data Availability

The authors declare that the data for this research are available from the correspondence authors upon reasonable request.
